# Patient expectations of podiatric surgery in the United Kingdom

**DOI:** 10.1186/1757-1146-4-27

**Published:** 2011-12-06

**Authors:** Antony N Wilkinson, Anthony J Maher

**Affiliations:** 1 Department of Podiatric Surgery, Doncaster and Bassetlaw Hospitals NHS Foundation Trust, Cantley Health Centre, Middleham Road, Cantley Doncaster, South Yorkshire, DN4 6ED, UK; 2 Department of Podiatric Surgery, Park House Health and Social Care Centre, 61 Burton Road, Carlton, Nottingham, NG4 3DQ, UK

## Abstract

**Background:**

Patient expectations can be difficult to conceptualise and are liable to change with time, health and environmental factors. Patient expectation is known to influence satisfaction, however little is known about the expectations of patients attending for podiatric surgery. This paper will explore the expectations of a large cohort of patients undergoing elective foot surgery.

**Methods:**

The UK based podiatric audit of surgery and clinical outcome measurement (PASCOM) audit system was applied to a consecutive cohort of patients undergoing elective podiatric surgery in Doncaster, South Yorkshire between 2004 and 2010. Data was collected relating to the surgical episode and patient expectations. A patient questionnaire was administered at 6 months post intervention.

**Results:**

A total of 2910 unique surgical admissions were completed and satisfaction questionnaires were returned by 1869 patients. A total of 1430 patients answered question 1 which relates to patient expectations. Pain relief was the most frequent expectation with 1191 counts (52.3%), while footwear and mobility accounted for 16.6% and 16.4% respectively. Cosmesis counts occurred less commonly; 12.2%. 709 patients (49.6%) stated only a single expectation, 599 patients (41.9%) stated two expectations, 114 patients (8%) stated three expectations and 7 patients (0.5%) stated 4 expectations. Pain relief was the dominant expectation accounting for 515 counts (72.6%) of patients who provided only one response.

**Conclusions:**

This paper demonstrates the expectations of a large cohort of podiatric surgery patients. For the most part patients expect pain relief, improved mobility and improved shoe fitting, while a small number of patients also expect a cosmetic improvement. Further research is required to determine the relationship between patient expectation and health related quality of life, and to determine whether podiatric surgery is successful in addressing the expectations of patients.

## Background

Within healthcare there has, in recent years, been a shift in research and audit towards the assessment of patient expectations, satisfaction and outcomes, as opposed to pure clinical measurements [1]. In the United Kingdom, the sea change can be traced back to 1983 when the National Health Service (NHS) Management Enquiry condemned the NHS for its failure to use market research techniques to evaluate service provision [2].

Patient expectations are related to satisfaction and satisfaction itself is best considered a consequence of successfully meeting a patient's expectations. Patient expectations can be difficult to conceptualise and are liable to change with time, health and environmental factors. Accepting the variability of patient expectation, satisfaction cannot be assessed in isolation without reference to the pre treatment expectations [3,4].

Patient expectations may directly influence satisfaction with the service provided. In evaluating satisfaction with a primary care out of hours services, Mckinley et al state that patients who receive the care they hoped for were more satisfied [5]. It is recognised that patient expectations are an important independent predictor of success or failure of total joint arthroplasty [6] and high but realistic expectations are associated with improved orthopaedic surgery outcomes [7-12]. The reverse is also true, in that unrealistically high expectations may adversely influence the outcome of surgery [7,13,14].

Within podiatric surgery authors have reported patient satisfaction as an outcome of intervention ([15-20]. Strangely though, there is little comment about patient expectations prior to elective foot surgery. A Medline search reveals only two previous papers expressly considering patient expectation in the context of hallux valgus surgery [21,22].

David Tollafield and Gavin Rudge developed a patient satisfaction questionnaire (PSQ) in the 1990's expressly for use in podiatric surgery [23,24]. The PSQ was developed as a component of the Podiatric Audit of Surgery and Clinical Outcome Measures (PASCOM) instrument which is an ongoing audit project in the United Kingdom tasked with data collection in podiatry. The system initially collected surgical activity data, including satisfaction scores through a Microsoft Access database which was analysed both locally and nationally [18,23].

The Patient satisfaction questionnaire (PSQ) asks a series of between 10 and 14 questions relating to a patient's experience of an episode of care (Additional File 1). There are a number of possible weighted answers for questions 2 to 10 and the scores for each are totalled with a maximum possible score of 100 and a minimum possible score of 0. The total score (PATSAT score) is said to be a reflection of the patient's satisfaction with their surgery experience [23-25].

Question one of the PSQ sits apart from its neighbours because it requires a free text response from the patient. Additionally there is no scoring or weight attached to it and as such question one is excluded from the summary scoring playing no part in the final PATSAT score. There has been surprisingly little mention of question one in podiatric surgery reports. This paper will utilise question one of the PSQ to explore the aims and expectations of a large cohort of patients undergoing Podiatric Surgery.

## Methods

The PASCOM audit system was applied to a consecutive cohort of patients undergoing elective podiatric surgery in Doncaster, South Yorkshire between 2004 and 2010. The PSQ Questionnaire was completed at 6 months post operation by all patients returning for a final check appointment. For the purposes of this study, there were no selection criteria beyond having undergone elective podiatric surgery and completed the PSQ.

The questionnaire was typically completed in the outpatient waiting room either immediately before or immediately following a clinic consultation. The questionnaires were entered onto an Access database by a team of healthcare assistants. The free text answers to question one were stored within an Access table.

The data was exported to an Excel spreadsheet for further analysis. A qualitative approach was adopted in order to categorise answers to Question one. An initial review of the free text answers identified four key responses which occurred repeatedly. These responses were labelled: Pain; Shoe Fitting; Activity; Cosmesis. Within each of these responses a number of key words were identified, these are listed in Table 1. The individual answers to question one were then reviewed again. A number of void answers were identified and discarded. The remainder were then placed in each of the four responses and an additional fifth theme; 'Other', which was used for miscellaneous answers. A large number of patients stated multiple expectations and so key words from any of the four response categories were determined and counted accordingly for each patient. The data relating to responses was categorical and so descriptive statistics were applied throughout.

**Table 1:** Patient expectation responses and example descriptors

Pain	Mobility	Shoe fitting	Cosmesis
Pain free/relief/reduced	Past times	Wear normal shoes	Deformity
Relief	Walking -easier	Stylish footwear/shoes	Ugly
Discomfort	Walking - better	Better fitting shoes	A straight(er) toe(s)
Comfort	Walking correctly	Shoe fit	Alignment
Rubbing	Dancing	Comfortable shoes	Lump/Bump
Irritation	Running	Wear a full shoe	Unsightly
Throbbing	Movement	Wear shoes properly	Nicer looking
Ache	Mobility		Removal of...
			Curled
			Crossing over
			Cosmetic

## Results

Between January 2004 and September 2010, a total of 2910 unique surgical admissions were entered onto the PASCOM audit database. The majority of patients were female (83.3%) and the age range was 14 - 95 years. The admissions gave rise to 5312 surgical procedures. All admissions were for day care surgery and the majority (99%) were performed under local anaesthesia. Seven podiatric surgeons and one podiatrist (non incision nail surgery procedures) contributed to the database.

Not all patients who underwent surgery subsequently completed the PSQ questionnaire, as a result a total of 1869 questionnaires were returned with the remaining 35.8% unaccounted for. As stated above, question one requires a text input. A total of 1458 returned questionnaires had some kind of input or wording entered for question one, while 411 were blank. A further 28 questionnaires were null and void where patient's had used the text box to make an unrelated comment or ask a question. This left a total of 1430 questionnaires available for further analysis.

Table 2 lists the number of counts recorded for expectations within each of the 5 response categories. A total of 2278 counts were recorded, 1191 counts (52.3%) were recorded for the Pain response while footwear and mobility accounted for 16.6% and 16.4% respectively. Cosmesis counts occurred less commonly accounting for 12.2%. A small number of counts (2.5%) did not fit comfortably into any of the 4 main categories.

**Table 2:** Total counts for each response (percentage of all themes).

Count	Pain	Mobility	Shoe fitting	Cosmesis	Other
Yes	1190 (52.2%)	374 (16.4%)	379(16.6%)	277 (12.2%)	57 (2.6%)

No	239	1056	1153	1051	1373

Total response counts = 2640. Total cohort = n.1430.

709 patients (49.6%) entered only a single expectation in the question one text box. 599 patients (41.9%) entered two expectations, 114 patients (8%) entered three expectations and 7 patients (0.5%) entered 4 expectations. Table 3 presents a summary of the responses for patients who entered only a single expectation. Again, pain relief was the dominant expectation accounting for 515 (72.6%) patients who provided only one response. Although cosmesis was the least common response overall, it was the second most common singular expectation accounting for 77 (5.4%) responses.

**Table 3:** Summary of counts for patients reporting a single expectation and percentage of total cohort (n = 1430)

Singular complaints	Count	Percentage
Pain	515	36.1%
Cosmesis	77	5.4%
Other	45	3.1%
Mobility	37	2.6%
Shoes	35	2.4%

**Total**	**709**	

Owing to the predominance of pain responses, we chose to further evaluate all questionnaires where an expectation of pain relief was recorded (Table 4). Pain responses were found in 1190 (83%) of the 1430 returned questionnaires. 36.1% of the cohort expected nothing more than pain relief, 16.6% expected pain relief and improved mobility, while 14.2% expected pain relief, and improved shoe fitting. The remaining 17.2% of patients who expected pain relief also had a number of additional expectations as detailed in Table 4.

**Table 4:** Summary of all pain response combinations and percentage of total cohort (n = 1430)

Pain response combinations	Count	Percentage
**Total Pain Counts (any combination)**	**1190**	**83%**

pain	515	36.1%
Pain; mobility	238	16.6%
Pain; shoes	203	14.2%
Pain; cosmesis	107	7.5%
Pain; mobility; shoes	58	4.1%
Pain; cosmesis; shoes	36	2.5%
Pain; mobility; cosmesis	18	1.3%
Pain; mobility; cosmesis; shoes	7	0.5%
Pain; other	6	0.42%
Pain, cosmesis, other	2	0.1%

## Discussion

The current paper has summarised for the first time, the expectations of patients attending for podiatric surgery. By far the most important expectation was for pain relief with 83% of patients referring to pain and 36.1% of patients expecting nothing more than pain relief. Expectations relating to mobility and shoe fitting were also important considerations while cosmesis was a relatively uncommon expectation. These findings are not dissimilar to those of Schneider and Knhar who found that the main expectations following hallux valgus surgery were to be pain free and able to wear a normal shoe [22]. The small number of counts for cosmesis is perhaps surprising given that most podiatric surgery procedures attempt to correct deformity. Radl et al, on the contrary, argue that cosmesis is actually a significant concern in hallux valgus surgery as opposed to many other orthopaedic procedures which address joint pain without consideration of cosmesis [26]. The current study did not assess expectation in relation to specific diagnoses but hallux valgus surgery accounted for 2162 (40%) procedures.

It is interesting to note that for the most part, patient expectations fell into four clearly defined responses (pain relief, shoe fitting, mobility and cosmesis) and that these responses are not dissimilar to the domains measured by regional measures of health related quality of life. For example two foot health instruments; the Foot Health Status Questionnaire (FHSQ)[27]; and the Manchester Oxford Foot questionnaire (MOXFQ)[28], both include domains relevant to pain, mobility and foot wear while the MOXFQ additionally makes specific reference to cosmesis. The current paper provides further support for the validity of the quality of life domains measured by both the FHSQ and MOXFQ.

Increasingly clinicians are relying on patient reported outcome measures which provide a validated and objective method of assessing a patient's health related quality of life prior to and following intervention. These instruments provide an invaluable insight into the impact of a given foot pathology on a patient's quality of life and in doing so may indirectly measure a patient's expectations and satisfaction. Bennet stated that health related quality of life (HRQOL) should be measured in preference to patient satisfaction [27]. On the contrary, Carr et al argue that HRQOL is actually defined as the gap between expectation of health and the patient's experience of health [4,29]. Patient expectations are then related to and may influence HRQOL, yet none of the current HRQOL instruments ask the question; 'what do you hope to gain from your treatment?' If patient expectations of healthcare are not considered, the planning of treatment appropriate to the patient's needs could be compromised.

Carr et al go further stating that HRQOL instruments cannot distinguish between changes in experience of disease and changes in expectation of health [4]. Patients will compare their personal experience and the experience of others to their own expectations of health when completing a HRQOL instrument [4,5]; The point where a given HRQOL scores start (prior to treatment) and finish (following intervention) is influenced by patient expectation and so if such instruments are used to study outcomes, arguably patient expectations must also be recorded [4].

Measurement of patient expectations and satisfaction is, one could argue a more personal or qualitative approach to assessing outcomes. Recording patient expectations of treatment allows clinicians' to better understand the impact of a disease process from the patient's perspective. This understanding may then be used to guide treatment planning. There is evidence that patient expectations are important in determining the success or failure of intervention and so expectations should arguably be determined prior to intervention. Patient expectations as presented during the initial consultation may actually influence treatment decisions although doctors perceptions have been found to be more important than patient expectation in determining treatment [30]. Therefore disparity between clinical perception of a patient's expectation of treatment and the patient's actual expectation may be a cause of poor outcomes [30].

It cannot be denied that question one of the PSQ offers patients an opportunity to describe their expectations. It is though important to note that this determination of patient expectation only occurs retrospectively. The patient is asked to consider their expectations sometime after treatment. This creates a significant methodological problem. However Kadezielski et al found that pre operative expectations did not correlate with post operative satisfaction in the context of carpal tunnel surgery [7]. Nonetheless, the current study cannot determine what effect treatment, recovery and overall perioperative experience have on how the patient answers question one.

We do know from previous work that patient expectations are at least partly a consequence of prior experience, the experience of others, environmental factors, personality type and the treatment in itself [4,26]. Similarly others have found an association between poor outcomes and unrealistic expectations [5,7,13,14]. In the current study, we did not attempt to quantify whether patient expectations were unrealistic. Rather we have presented expectations as found. This paper also chose to review patient expectations across all podiatric surgery procedures as a single cohort whereas previous authors have concentrated on hallux valgus surgery alone [21,22,26]. There may be some value in analysing expectations relating to specific diagnoses, Delgado et al found that expectations vary depending on the presenting disease process in the setting of primary care [31].

Data collection for this study started before the introduction of patient reported outcomes into routine podiatric surgery practice in the UK. Analysing patient expectation in relation to pre operative HRQOL scores and measuring patient satisfaction in relation to post operative HRQOL scores would be a valuable endeavour. We cannot, at this time, be certain of what impact (if any) patient expectation has on health related quality of life in the context of foot surgery. Although we now have a basic understanding of expectation we do not know what factors, such as quality of life or surgical complications, may influence the clinician's ability to meet a patient's expectations and further work is required to determine why expectations have or have not been met and to determine the impact multiple expectations may have on surgical outcomes.

There were a number of weaknesses with the study design. Perhaps most significant of these is retrospective data collection. Patients were asked to describe their pre operative expectations of surgery, 6 months following treatment. There was no attempt to correlate this finding with a pre operative measure of expectation. Retrospective data collection also resulted in a significant loss to follow up. During the study period 2910 patients attended for surgery but only 1869 questionnaires were returned. We do not know what effect this loss to follow up had on the actual expectation responses.

Compounding the loss to follow up, the design of the questionnaire itself resulted in a further data loss with only 1430 patients completing question 1. This data loss could be minimised by formatting question 1 as per the remaining PSQ questions; utilising a list of responses and tick boxes as opposed to a free text.

## Conclusions

This paper demonstrates the expectations of a large cohort of podiatric surgery patients. For the most part patients expect pain relief, improved mobility and improved shoe fitting, while a small number of patients also expect a cosmetic improvement. Further research is required to determine the relationship between patient expectation and health related quality of life, and to determine whether podiatric surgery is successful in addressing the expectations of patients.

## Competing interests

AJM is a current member of the PASCOM working party of the Society of Chiropodists and Podiatrists, UK and is directly involved with developmental work relating to outcome measurement. Neither author received a financial reward for the production of this manuscript.

## Authors' contributions

ANW took responsibility for the implementation of PASCOM in Doncaster, England and over saw data collection. The paper was jointly devised by AJM and ANW, AJM prepared the manuscript and both authors reviewed and edited the final manuscript prior to submission.

## Supplementary Material

Additional file 1 **Patient Satisfaction Questionnaire**. Click here for file
